# Complete Genome Sequence of Leptospira kmetyi LS 001/16, Isolated from a Soil Sample Associated with a Leptospirosis Patient in Kelantan, Malaysia

**DOI:** 10.1128/MRA.00015-19

**Published:** 2019-07-11

**Authors:** Nik Yusnoraini Yusof, Farhana Muhammad Yusoff, Salwani Muhammad Harish, Muhammad Nuramin Ahmad, Muhammad Fazli Khalid, Fauziah Mohd Nor, Nabilah Ismail, Ismail Aziah

**Affiliations:** a Institute for Research in Molecular Medicine (INFORMM), Health Campus, Universiti Sains Malaysia, Kubang Kerian, Kelantan, Malaysia; b Kelantan Public Health Laboratory, Kota Bharu, Kelantan, Malaysia; c Department of Medical Microbiology and Parasitology, School of Medical Sciences, Health Campus, Universiti Sains Malaysia, Kubang Kerian, Kelantan, Malaysia; University of Maryland School of Medicine

## Abstract

The Gram-negative pathogenic spirochetal bacteria *Leptospira* spp. cause leptospirosis in humans and livestock animals. Leptospira kmetyi strain LS 001/16 was isolated from a soil sample associated with a leptospirosis patient in Kelantan, which is among the states in Malaysia with a high reported number of disease cases. Here, we report the complete genome sequence of Leptospira kmetyi strain LS 001/16.

## ANNOUNCEMENT

Leptospirosis is a zoonotic infectious disease caused by bacteria in the genus *Leptospira* (1). Leptospires are widespread due to their ability to infect a variety of animal species, including humans, and also their ability to survive outside the host under appropriate conditions (2, 3). Several outbreaks of leptospirosis have happened following natural disasters, such as heavy rainfall and frequent flooding, as well as activities in recreational water (4–7). The disease is usually transmitted through the urine of infected or carrier animals and contaminated water, soil, and mud (8). Transmission can occur through direct or indirect contact with infected animals or their secretions (6, 7). In 1907, *Leptospira* bacteria were first found in the kidney tissue of a deceased leptospirosis victim who was diagnosed as having “yellow fever.” Here, we report an isolate that was obtained from a soil sample in a leptospirosis patient locality in Pasir Mas, Kelantan, Malaysia. In brief, the soil sample was mixed with 30 ml of sterile water and was filtered using a 0.22-μm Nalgene filter unit. Then, it was transferred to Ellinghausen-McCullough-Johnson-Harris (EMJH) liquid medium and incubated at 30°C with 25 rpm for 28 days. PCRs were performed for species identification using primer pairs to the *Bak2* gene and *lipL32* gene (9).

Genomic DNA was extracted using a Genomic-tip 100/G and genomic buffer set (Qiagen, USA), following the protocol by the manufacturer. A 10-kb library was prepared using a SMRTbell template prep kit 1.0 (Pacific Biosciences, Menlo Park, CA) and sequenced on one single-molecule real-time (SMRT) II cell. The whole-genome *de novo* assembly was carried out by Hierarchical Genome Assembly Process 3.0 (HGAP 3.0) (10).

The assembly was improved with error correction by Quiver v1 in SMRT Portal v2.3.0. The assembly was improved with error correction by Quiver v1 in SMRT portal v2.3.0. The polymerase reads were quality filtered and trimmed to acquire a high-quality region, with a minimum subread length, a minimum polymerase read quality, and a minimum polymerase read length of 500 bp, 0.80, and 1,000 bp, respectively. The sequencing coverage based on the subreads was ∼159×. The *N*_50_ value of raw sequences is 3,927,288 bp. The final assembly of the genome comprised 2 contigs with a total contig size of 4,416,922 bp with two chromosomes with sizes of 3.93 Mb and 0.49 Mb. The G+C contents of chromosome 1 and chromosome 2 are 44.83% and 44.6%, respectively, with a total of 3,059 protein-coding sequences, 37 tRNAs, and 6 rRNAs. Genome prediction and annotation were done using CLgenomics (http://data.chunlab.com/software/clgenomics). The genome contains 2,019 (66%) protein-coding sequences, and their biological function was assigned based on the Clusters of Orthologous Groups (COG) database (8). The prediction revealed a total of 34% genes with unknown function that will be valuable for further investigation that might contribute to our understanding of strain pathogenicity.

### Data availability.

The genome sequence has been deposited at GenBank under the accession no. CP033614 and CP033615 (BioProject accession no. PRJNA504370 and BioSample accession no. SAMN10390430). The SRA accession number for the raw data is SRR8706750.
